# Resilience-aware MLOps for AI-based medical diagnostic system

**DOI:** 10.3389/fpubh.2024.1342937

**Published:** 2024-03-27

**Authors:** Viacheslav Moskalenko, Vyacheslav Kharchenko

**Affiliations:** ^1^Department of Computer Science, Faculty of Electronics and Information Technologies, Sumy State University, Sumy, Ukraine; ^2^Department of Computer Systems, Network and Cybersecurity, Faculty of Radio-Electronics, Computer Systems and Infocommunications, National Aerospace University “KhAI”, Kharkiv, Ukraine

**Keywords:** MLOps, medical diagnosis, image recognition, robustness, resilience

## Abstract

**Background:**

The healthcare sector demands a higher degree of responsibility, trustworthiness, and accountability when implementing Artificial Intelligence (AI) systems. Machine learning operations (MLOps) for AI-based medical diagnostic systems are primarily focused on aspects such as data quality and confidentiality, bias reduction, model deployment, performance monitoring, and continuous improvement. However, so far, MLOps techniques do not take into account the need to provide resilience to disturbances such as adversarial attacks, including fault injections, and drift, including out-of-distribution. This article is concerned with the MLOps methodology that incorporates the steps necessary to increase the resilience of an AI-based medical diagnostic system against various kinds of disruptive influences.

**Methods:**

*Post-hoc* resilience optimization, *post-hoc* predictive uncertainty calibration, uncertainty monitoring, and graceful degradation are incorporated as additional stages in MLOps. To optimize the resilience of the AI based medical diagnostic system, additional components in the form of adapters and meta-adapters are utilized. These components are fine-tuned during meta-training based on the results of adaptation to synthetic disturbances. Furthermore, an additional model is introduced for *post-hoc* calibration of predictive uncertainty. This model is trained using both in-distribution and out-of-distribution data to refine predictive confidence during the inference mode.

**Results:**

The structure of resilience-aware MLOps for medical diagnostic systems has been proposed. Experimentally confirmed increase of robustness and speed of adaptation for medical image recognition system during several intervals of the system’s life cycle due to the use of resilience optimization and uncertainty calibration stages. The experiments were performed on the DermaMNIST dataset, BloodMNIST and PathMNIST. ResNet-18 as a representative of convolutional networks and MedViT-T as a representative of visual transformers are considered. It is worth noting that transformers exhibited lower resilience than convolutional networks, although this observation may be attributed to potential imperfections in the architecture of adapters and meta-adapters.

**Сonclusion:**

The main novelty of the suggested resilience-aware MLOps methodology and structure lie in the separating possibilities and activities on creating a basic model for normal operating conditions and ensuring its resilience and trustworthiness. This is significant for the medical applications as the developer of the basic model should devote more time to comprehending medical field and the diagnostic task at hand, rather than specializing in system resilience. Resilience optimization increases robustness to disturbances and speed of adaptation. Calibrated confidences ensure the recognition of a portion of unabsorbed disturbances to mitigate their impact, thereby enhancing trustworthiness.

## 1 Introduction

### 1.1 Motivation

The advent of Artificial Intelligence (AI) in healthcare has opened new horizons in medical diagnostics, offering more precise, efficient, and rapid techniques for detecting a wide range of diseases. However, the critical nature of healthcare imposes strict requirements on AI-based diagnostic systems, necessitating robust performance, high reliability, and stringent data security measures. Despite the attention to quality, security, and performance in traditional Machine Learning Operations (MLOps), an overlooked aspect remains—resilience to disturbances.

In healthcare applications, AI-based systems are exposed to numerous disturbances that can significantly impact their effectiveness. These disturbances may range from adversarial attacks designed to manipulate model outputs, to fault injections that can undermine system integrity, and to drift phenomena where the model’s performance degrades due to changing patterns in data distribution. Conventional MLOps methodologies focus extensively on data quality, model performance, and security but do not adequately address these resilience challenges. Given the potentially life-altering decisions that AI-based medical diagnostic systems are entrusted with, a lack of resilience can have severe consequences, including inaccurate diagnoses and, consequently, improper treatment plans.

Moreover, the unique characteristics of the healthcare domain such as patient-specific variabilities, heterogeneous data sources, and strict regulatory constraints introduce distinctive kinds of disturbances that are not commonly observed in other sectors. Therefore, a “one-size-fits-all” approach from other domains cannot be directly applied here.

The ability of a system to be resilient—to absorb, detect, and adapt to disturbances—is particularly crucial in high-stakes environments like healthcare. A resilient-aware MLOps framework for AI-based medical diagnostic systems would not only improve their robustness but would also enhance trust among clinicians, healthcare providers, and patients, thus accelerating the adoption rate of AI in healthcare.

In light of these challenges and opportunities, this study aims to enrich MLOps methodology by incorporating resilience as a fundamental component. By identifying characteristic disturbances in healthcare and developing methods to ensure resilience, this study endeavors to elevate the reliability and trustworthiness of AI-based medical diagnostic systems, making them better equipped to provide quality healthcare solutions in dynamic and unpredictable environments.

### 1.2 State-of-the-art

The evolution of AI in healthcare has led to various significant advancements, many of which are integrated into existing MLOps frameworks (1). A plethora of research exists, focusing on improving data quality, model training, evaluation, and deployment in the healthcare domain (2, 3).

Machine learning operations have gained momentum in healthcare due to their potential to streamline the development, deployment, and maintenance of machine learning models (4). Several studies have delved into the unique requirements and challenges that healthcare poses to the MLOps methodology, such as patient data confidentiality (5), bias reduction (6), and compliance with health regulations like HIPAA (7). However, most existing MLOps frameworks are designed to ensure efficient operation rather than resilience to the various disturbances that healthcare environments may present.

The concept of resilience in AI systems is not new and has been examined across various fields, including cybersecurity, manufacturing, and even autonomous vehicles. Techniques like adversarial training (8, 9), robust optimization (10), and uncertainty quantification have been employed to improve resilience (11). The paper (12) proposes the concept of Secure Machine Learning Operations paradigm, but without proposals for combining different methods and aspects of protecting the same AI system from different threats. The issue of resolving the incompatibility of the selected approaches in the tasks of ensuring the resilience and efficiency of the AI system is not considered. The vast majority of researchers consider each type of disturbance for AI systems separately, and the question of compatibility of methods for ensuring resilience to each of these disturbances remains under-researched (13–15). In addition, although these methods provide a certain degree of perturbation absorption, they often do not ensure rapid adaptation and evolution in response to changing conditions.

Despite the abundance of work in MLOps, resilience, and AI-based medical diagnostics separately, there is a conspicuous absence of research focusing on integrating resilience into MLOps frameworks specifically designed for medical diagnostic systems. This gap highlights the need for a holistic approach that combines these elements to ensure not just efficiency and reliability, but also resilience against the myriad disturbances that these systems may encounter.

### 1.3 Objectives and contributions

The aim of this study is to develop a new MLOps methodology that ensures the resilience of a medical diagnostic system to such negative factors as adversarial attacks, failure injection, drift, and out-of-distribution of data. The key objectives are as follows:

- analysis of resilience issue of MLOps for healthcare;
- analysis of methods of ensuring the resilience of AI-systems;
- develop resilienсe-aware MLOps architecture for Medical Diagnostic Systems; and
- experimentally confirm the advantages of resilience-aware MLOps compared to the conventional approach.

Structurally, the work consists of the following sections. The analysis of methods of ensuring the resilience of MLOps and resilienсe-aware MLOps architecture for Medical Diagnostic Systems are presented in Section 2. The Section 3 describes the experimental results of testing and comparition of the proposed resilience-aware MLOps and Conventional MLOps. The research results are discussed in the Section 4. The Section 5 concludes the paper and describes the directions of future research.

The main contribution of the research includes architectures of resilience-aware MLOps for Medical Diagnostic Systems. In addition, the results of the comparison between the traditional and the proposed MLOps on the MedMNIST datasets are analyzed. It has been experimentally proven that the addition of resilience optimization, predictive uncertainty calibration, uncertainty monitoring, and graceful degradation makes a positive contribution to the robustness and performance recovery of a medical diagnostic system.

## 2 Architecting resilient MLOps-based medical diagnostic system

### 2.1 Resilience issue of MLOps for healthcare

**Figure 1:**
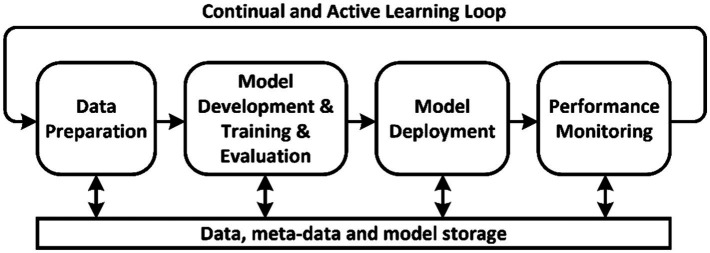
Basic stages of conventional MLOps.

Machine learning operations is the process of automating the lifecycle of machine learning models. It involves four main stages (Figure 1) (1, 2):

1. Data Preparation—gathering, cleaning, and transforming data for further model training.
2. Model Development, Training and Evaluation—building the architecture, training, and testing the model on prepared data.
3. Model Deployment—integrating the trained model into a production environment.
4. Performance Monitoring—tracking the model’s metrics in operation and providing feedback to the data preparation stage.

Conventional approaches to MLOps often do not pay enough attention to the resilience of machine learning systems to the perturbations inherent in the medical domain. They do not focus on absorbing and detecting disturbances and adapting to them quickly. However, for medical applications, these aspects are extremely important, as human lives depend on the recommendations of ML systems. Disturbances in an intelligent system can be caused by both intentional attacks and natural causes. Examples of natural disturbances include noise in the data, sudden hardware faults (memory faults), and data drift over time (16). Intentional attacks can also include fault injections and data manipulation in the form of so-called adversarial attacks.

Drift is particularly relevant to the healthcare industry, as disease patterns can change due to new strains of viruses and bacteria and disease patterns can change due to changes in treatment protocols (17). Data characteristics may also change due to improvements in medical equipment, changes in data collection methods, and changes in demographics. In addition, the emergence of new data, the identification of previously unknown relationships and factors, and the refinement of disease taxonomies are additional sources of concept drift. The main problem with drift adaptation is the delay in the arrival of labeled data after drift occurs, so the ability to quickly adapt to a small amount of labeled data is a very relevant property.

Adversarial attacks in AI-based medical diagnostic systems refer to deliberate manipulations of input data (such as medical images) designed to deceive AI algorithms (18). These attacks exploit vulnerabilities in the AI’s learning process, where slightly perturbed images, indistinguishable to the human eye, can lead to incorrect diagnoses or assessments. The source of these attacks can vary, ranging from external threats aiming to undermine the system’s reliability to internal errors in training data or algorithm design. However, to protect against adversarial attacks, the initial development of models and methods for training intelligent diagnostic systems may be complicated by the need to investigate the compatibility of various methods for enhancing the robustness and resilience of AI systems (19).

In the computational environment of an intelligent medical diagnostic system, malicious faults, commonly known as fault injections, can pose significant threats. These deliberate disruptions can be executed in various forms, targeting different components of the system. For instance, one notable type of attack is the “row hammer” attack on memory (20). This involves repeatedly accessing a row of memory cells to induce bit flips in adjacent rows, potentially corrupting data or causing system crashes. The existing efforts to increase fault tolerance and the adaptation rate to a certain amount of faults may not be compatible with protection against other types of disturbances.

Thus, AI algorithms used in the healthcare industry have numerous vulnerabilities that traditional MLOps approaches do not focus on. Therefore, a promising direction for the development of AI-based medical diagnostic systems is the use of MLOps with elements to ensure resilience to disturbances.

### 2.2 Methods of ensuring the resilience of AI systems

Table 1 shows the approaches to ensure the resilience to adversarial attacks, fault injection, and concept drifts for AI system. The ability to absorb disturbances (robustness), graceful degradation due to the impact of disturbances that could not be absorbed, and rapid adaptation to new disturbances are considered to be the key features of resilient system. “Graceful degradation” refers to a system being pre-configured with an organized set of less functional states. These states represent acceptable compromises between functionality, performance, and cost-effectiveness.

**Table 1:** Approaches to ensure the resilience of AI-systems.

Disturbance source	Resilience capabilities		
	Robustness	Graceful degradation	Adaptation
Drift, Out-of-distribution data	Out-of-domain generalization (21); Ensemble selection (21)	Detecting adversarial examples (22), drift, out-of-distribution data (23), and faults (24); AI Explanation mechanism (25); Zero-shot learning (26); Switch to simpler model (27) or Reduce prediction granularity (28)	Active Learning (29); Continual Learning (30); Few-Shot Learning (31).
Adversarial attack	Data encryption and sanitization (32); Gradient Masking (33); and Robustness Optimization (10)		
Fault injection	Fault masking (34); Explicit redundancy (35); and Error detection and correction (24)		

Implementation of out-of-domain generalization through domain randomization or domain augmentation increases model robustness to limited shifts in data distribution (21). Dynamic adjustment of model weights in an ensemble can mitigate a certain level of concept drift (32). In addition, the ability to detect drift or out-of-distribution data can provide graceful degradation by delegating control to a human or to an AI model that is more resistant to such a disturbance.

Robustness to adversarial attacks can be enhanced by protecting training data or restricting access to knowledge about the AI model. The protection of training data is usually achieved through data encryption and sanitization (36). The incorporation of randomization or non-differentiable input transformations into the model provides gradient masking to counteract adversarial evasion attacks (33). Moreover, adding different regularization and training on adversarial samples provides robust optimization (10). However, detecting adversarial examples can be an effective mechanism for graceful degradation by delegating control to humans or automatically switching to models that are more resilient to such a perturbation (22).

Redundancy in the form of N-versioning of AI model or duplication of critical neurons is the most common way to ensure robustness to faults (fault tolerance) (35). However, training under noise added to the gradient or neurons weights can also help to reduce the importance of individual neurons by providing fault masking, i.e., eliminating their influence (34). However, the use of error detection mechanisms for model weights can be combined with error correction mechanism or with downloading an uncorrupted version of the weights (24). If an error in the weights causes abnormal distortions in the feature space, it can increase predictive uncertainty and require a delegation of control to a human or switching to another model or model branch (27, 28).

Estimating model uncertainty is a useful function to identify the negative impact of any destructive influences on the AI system. However, by default, conventional AI model do not provide an efficient predictive uncertainty estimation and it requires calibration. In addition, detection of destructive disturbances affecting the AI model can be achieved through the mechanisms of AI explainability.

Graceful degradation can occur by increasing the resource consumption for disturbance processing, for example, by delegating control to an expert or large model. An expert can be reinforced by an AI explanation algorithm, while a large model is used with auxiliary semantic information (i.e., Zero-shot learning) (26). Switching to a simpler model can also be viewed as a graceful degradation, as a simple model is generally less sensitive to disturbances in the data, but produces more coarse or abstract predictions (27, 28).

Adaptation to disturbance typically occurs through retraining in the face of disturbance using Active Learning, Continuous Learning, and Few-Shot Learning methods (29–31). However, to increase the speed of adaptation, meta-learning and Parameter-Efficient Fine Tuning methods are widely used (37). In addition, meta-learning is also effective in optimizing robustness (38).

Annotation of training data for medical diagnostic system requires deep medical knowledge, while the knowledge is constantly expanding and updating. There is a need to integrate Active Learning in MLOps feedback to effectively use data and time of highly qualified experts (29). At the stage of training data preparation, an expert could annotate the most complex cases. Complicated cases where the AI system has the greatest uncertainty can be identified by a specified uncertainty indicator. The simplest and most logical way to measure uncertainty is to calculate Shannon’s Entropy Measure for classification model or using quantile regression for regression model.

Detection of out-of-distribution, concept drift, a certain fraction of adversarial attack and fault injection effects can be implemented by testing for exceeding the threshold value of the model uncertainty indicator. In this case, the threshold value of the uncertainty indicator can be defined as a 95% percentile on an augmented test or training dataset.

Thus, there are methods for implementing different resilience features for different disturbances. However, the vast majority of them require changing the model or learning algorithm, which complicates the responsibility separation in MLOps. In this case, making the AI system resilient will require additional research into the compatibility of different mechanisms for ensuring resilience to various disturbances and their mutual influence.

### 2.3 Resilienсe-aware MLOps architecture for medical diagnostic systems

Important principles in MLOps are the separation of responsibilities and collaboration between teams. Platform-level specialized solutions to ensure the resilience of any AI model delegates the updating and maintenance of this mechanism to a separate team of AI resilience experts. New MLOps stages for ensuring resilience aspects should be implemented as *post-hoc* procedures to maximize the separation of responsibilities.

**Figure 2:**
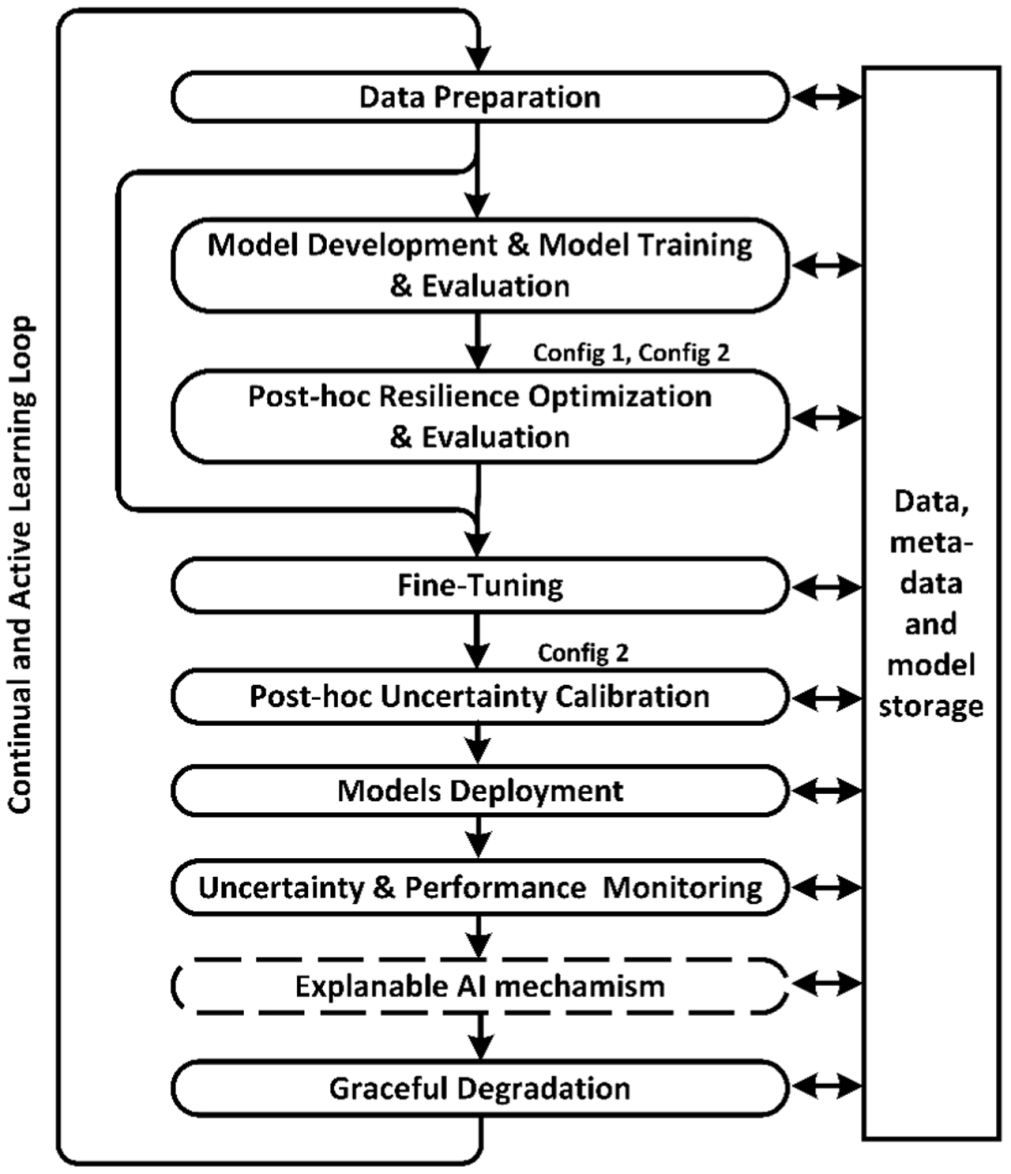
Basic stages of resilience-aware MLOps.

Figure 2 shows a diagram of the proposed resilience-aware MLOps, which additionally includes the stages of *Post-hoc* Resilience Optimization, *Post-hoc* Uncertainty Calibration, and Uncertainty Monitoring and Graceful Degradation. In addition to Uncertainty Monitoring, the Explainable AI mechanism can be used to assist decision-making by the human to whom control is delegated in case of uncertainty. The article (39) questions the necessity and adequacy of existing methods of explaining decisions, so the explanation mechanism will be excluded from further consideration, but for generality, the diagram shows this MLOps stage.

**Figure 3:**
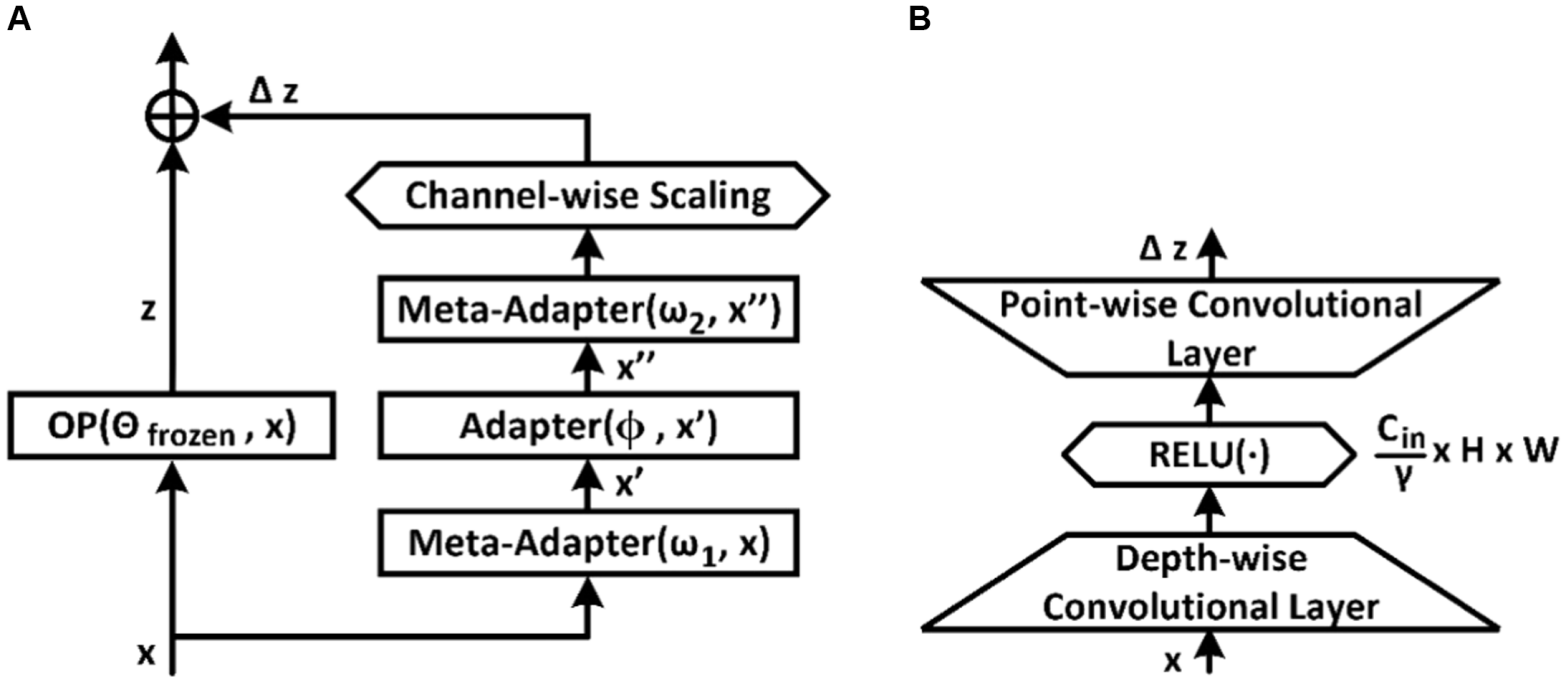
Parallel tuning scheme and tuner architectures. **(A)** Parallel tuning scheme for the frozen block; **(B)** Adapter or meta-adapter based on two-layer convolutional network with channel dimension down-sampling bottleneck.

At the stage of resilience optimization, it is proposed to attach computationally efficient (meta-) adapters to the original model in order to increase robustness and speed up the fine-tuning process (40). In this case, the weights of the original model remain frozen. The original model usually consists of certain blocks or modules, for example Convolutional Residual Block. To generalize, we will refer to these blocks as frozen operations and denote them as $\mathrm{OP}\left(x\right)$ . The parallel method of connecting an adapter to the frozen blocks of the model is the most convenient and versatile approach (Figure 3A). In this case, to ensure the properties of resilience, it is proposed to use three consecutive blocks of adapters at once, two of which are tuned during meta-training (40). To balance between different modules, we introduce a channel-wise scaling factor.

The adapter architectures depicted in Figure 3B are based on convolutional layers. The convolutional adapter shown in Figure 3B has a hyperparameter γ , which regulates channel compression by 1, 2, 4, or 8 times. However, adapters can also be implemented as a two-layer feed-forward network with a downward projection bottleneck or ResNet-like conversion.

The original model is trained on a dataset $D_{\mathrm{base}}=\left\{D_{\mathrm{base}}^{tr};D_{\mathrm{base}}^{\mathrm{val}}\right\}$ to perform the main task under known conditions. Resilience optimization involves generating a set of synthetic disturbance implementations $\left\{\tau_{i}|\;i=\bar{1,N}\right\}$ . As disturbances $\tau_{i}$ can be considered adversarial attacks, fault injection, or switching to a new task. In addition, it is necessary to provide datasets $D=\left\{D_{k}^{tr};|D_{k}^{\mathrm{val}};|k=\bar{1,K}\right\}$ , that solve other problems for K few-shot learning tasks, where fine-tuning data $D_{k}^{tr}$ is used in the fine-tuning stage and validation set $D_{k}^{\mathrm{val}}$ is used in the meta-update stage. There is also a given set of parameters θ , ϕ , ω , and W , where θ are parameters of a pretrained and frozen base AI model, ϕ and ω are adaptation parameters of AI model backbone, and W are task specified parameters (model head parameters). Head weights $W_{\mathrm{base}}$ for the main task are pre-trained on the data $D_{\mathrm{base}}$ . If we reject the specialization of different parameters of the AI model and denote the set of all parameters as $\Xi =\langle \theta ,\phi ,\omega ,W\rangle$ , then the process of meta-learning for direct maximization of the expected resilience criterion can be described by the formula:

(1) $$\Xi^{*}=\underset{\Xi }{\mathrm{argmax}}\underset{\tau_{i}∼p\left(\tau \right)}{E}\left[R_{\tau_{i}}\left(U\left(\Xi ,D\right)\right)\right]=\underset{\Xi }{\mathrm{argmax}}F\left(\Xi \right)$$

where U is an operator that combines disturbance generation and adaptation in T steps, which maps the current state of ϕ to new state of ϕ ;

$R_{\tau_{i}}$ is a function that calculates the value of the integral resilience indicator for $\tau_{i}$ disturbance implementation over model parameters ω during its adaptation and the test sample $D_{\tau_{i}}^{\mathrm{val}}$ .

(2) $$R_{\tau_{i}}=\frac{1}{P_{0}T}\overset{T}{\underset{t=1}{\sum }}P_{\tau_{i}}\left(\theta ,\omega ,\phi_{t},W_{t},D_{\tau_{i}}^{\mathrm{val}}\right)$$

where $P_{\tau_{i}}$ is a performance metric for current state of model parameters and evaluation data.

If we use the SGD stochastic gradient descent algorithm with T steps in the U operator and use gradient meta-update in the outer loop, we will get the algorithm shown in Figure 3. The meta-gradient estimation can be performed over the Gaussian-smoothed version of the outer loop objective, which is calculated by the formula (38).

(3) $$\nabla \underset{g∼N\left(0,I\right)}{E}\left[F\left(\Xi +\sigma g\right)\right]=\frac{1}{2\sigma }E\left[R\left(\Xi +\sigma g\right)-R\left(\Xi -\sigma g\right)\right]$$

**Figure 4:**
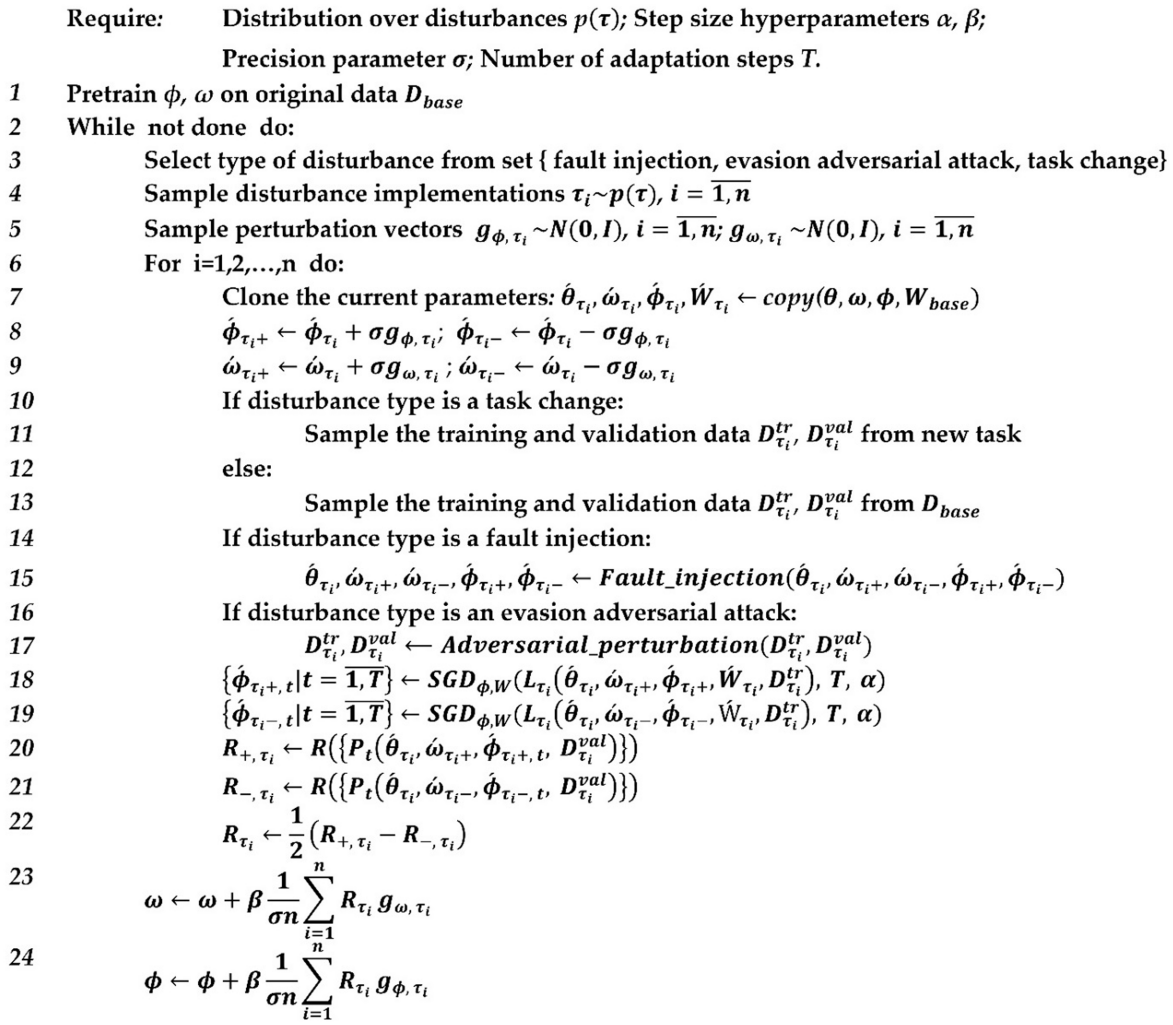
Pseudocode of model-agnostic meta-learning with evolution strategies for AI-system resilience optimization.

A perturbation vector *g* is formed for the meta-optimized parameters at the beginning of each meta-optimization iteration; the resulting algorithm will be as shown in Figure 4.

The analysis of Figure 4 shows that the type of disruptive influence does not change within a single meta-adaptation step. However, each meta-adaptation step begins with the selection of a disruptive influence type, followed by the generation of n implementations of the disruptive influence with a subsequent nested adaptation loop for each of them. Simultaneously combining disturbances may be ineffective. For example, after adding fault injection to the weights, we will have an outdated model, and applying adversarial attacks to it may be irrelevant.

The formation of adversarial samples is based on the $Adv:\mathrm{perturbation}\left(\right)Adv:\mathrm{perturbation}\left(\right)$ function. For differentiable models, FGSM attacks or PGD attacks can be used (36, 41). It is proposed to use adversarial attacks based on the search algorithm of the covariance matrix adaptation evolution strategy for non-differentiable models (39). The level of perturbation is limited by the $L_{\infty }$ -norm or $L_{0}L_{0}$ -norm. In this case, if the image is normalized by dividing pixel brightness by 255, then the specified disturbance level is also divided by 255.

The formation of fault injections is performed by the $\mathrm{Fault}:inj\left(\right)\mathrm{Fault}:inj\left(\right)$ (42). It is suggested to choose the most difficult fault type to absorb, which involves generating an inversion of a randomly selected bit (bit-flip injection) in the model weight. For differentiable models, it is suggested to pass the test dataset through the network and calculate the gradients, which can then be sorted by their absolute values. In the top-*k* weights with the highest gradient, one bit is inverted in a random position. The proportion of weights for which one random bit is inverted can be denoted as the fault rate.

Task change is needed to simulate concept drift and out-of-distribution. Forming a sample of other tasks can be done by randomizing the domain of the same task or by selecting tasks from relevant domains but sampling truly different tasks. These two approaches can also be combined.

Augmented versions of training samples can be used for improved calibration on in-distribution data. Out-of-distribution data can be generated from other datasets that do not share similar labels. Out-of-distribution data can be generated from other datasets that do not have semantically similar labels. One of the effective methods of generating Out-of-distribution is the use of Soft or Hard Brownian Offset Sampling with Autoencoders (43). Software libraries and examples of application to various data modalities are available for the Soft Brownian Offset algorithm.

The *post-hoc* calibration algorithm requires adding certain add-ons to the frozen model that are adjusted on the calibration data to reduce the discrepancy between the prediction confidence and the actual probability. Calibration Add-Ons for classification model based on Temperature Scaling, Platt Scaling, Isotonic Regression, Histogram Binning, Bayesian neural networks, ensembles, etc.

Active Learning is a widespread practice in the medical industry and in our MLOps diagram, it is part of the feedback loop. In the case of conventional MLOps, the base model is tuned on the new data, while in the case of resilienсe-aware MLOps, adapters are tuned. Re-training of the base model and resilience optimization can be performed after a certain predefined amount of new data has been accumulated since the last resilience optimization.

## 3 Experiments and results

### 3.1 Experimental setup

MedMNIST datasets contain annotated medical data samples for testing machine learning and artificial intelligence techniques in healthcare (44). Experimental research is proposed to be performed on these datasets. DermaMNIST dataset, which contains seven classes, will be considered as the main task dataset. The BloodMNIST and PathMNIST datasets will be used as data sources for few-shot learning tasks in the resilience optimization (meta-learning) process. In this case, a set of five classes will be used for few-shot learning tasks, which are randomly selected from the set of available classes ( $n_{\mathrm{way}}=5$ ). It is proposed to use 16 images per class (k_shot = 16), which are provided in mini-batches by four images (mini_batch_size = 4) during adaptation. Thus, the number of adaptation steps is T = (k_shot* $n_{\mathrm{way}}$ )/mini_batch_size = 20 iterations. The learning rate of the inner and outer loop of meta-learning are *α* = 0.001 and *β* = 0.0001, respectively. The maximum number of meta-iterations is 300. However, the Early Stopping algorithm is used to stop meta-learning, which terminates the execution if the criterion does not change for more than 10 consecutive iterations by more than 0.001. In this case, the convolutional network ResNet-18 and the visual transformer MedViT-T will be used as representatives of two main approaches to building a neural network architecture in the field of computer vision (45). In the case of ResNet-18, adapters and meta-adapters are connected in parallel to each ResBlock. In the case of MedViT-T, adapters and meta-adapters are connected in parallel to each Local Feed-Forward Network and Multi-Head Self-Attention Module.

Several configurations will be considered to illustrate the impact of additional stages of resilience-aware MLOps on the accuracy and speed of its recovery:

- Config 0—conventional MLOps with Fine-Tuning stage and Active Learning Feedback Loop;
- Config 1—upgraded Config 0 with Resilience Optimization stage; and
- Config 2—upgraded Config 1 with Predictive Uncertainty Calibration stage.

MedMNIST datasets contain training, validation, and test subsamples. To simplify the experiment and analyze the results, we will combine the validation and test samples into one test set and divide it into four parts. Each part of the test data is needed to simulate a part of the AI model’s life cycle. Let us consider four consecutive parts of the life cycle:

- Test 0—training (parameter optimization) of the AI model on the training set and testing the model on the first part of the test set, and selecting 10% of the test data points with the highest predictive uncertainty;
- Test 1—fine-tuning of the AI model on the selected data points from the previous test and testing the model on the second part of the test dataset under the disturbance, and selecting 10% of the test data points with the highest predictive uncertainty;
- Test 2—fine-tuning on the selected data points from the previous test and testing the model on the third part of the test dataset under the disturbance, and selecting 10% of the test data points with the highest predictive uncertainty; and
- Test 3—fine-tuning on selected test data points from the previous test and testing the model on the fourth part of the test data set under conditions of increased disturbance intensity.

In order to keep things simple, we assume that the graceful degradation is implemented as a decision rejection in case of uncertainty detection, i.e., if the entropy exceeds a threshold. We assume that control is transferred to a human, a more efficient model, or a preconfigured default procedure. Therefore, we will consider model accuracies calculated in two ways:

- ACC1 is the accuracy which counts rejected examples as false decisions; and
- ACC2 is the accuracy that does not take into account rejected examples.

Conventional MLOps reject decisions based on predictive confidence, while resource-aware MLOps reject decisions based on uncertainty.

For training adapters with meta-adapters, fault injection is carried out by selecting weights with the largest absolute gradient values. The proportion of modified weights is fault_rate = 0.1. For testing the resulting model, fault injection will be performed by random bit-flips in randomly selected weights, the proportion of which (fault_rate) are equals to 0.1 or 0.15.

The training of the tuners and meta-tuners involves generating adversarial samples using the FGSM algorithm with perturbation_level according to L∞ up to 3. However, to test the resulting model against adversarial attacks, the adversarial samples are generated using the CMA-ES algorithm with perturbation_level according to L∞-norm are equals to 3 or 5. The number of solution generation in the CMA-ES algorithm is set to 10 to reduce the computational cost of conducting experiments.

Instead of directly modeling different types of concept drift or novelty in the data, it is proposed to model the ability to quickly adapt to task changes, as this can be interpreted as the most difficult case of real concept drift. The preparation for the experiment involved adding adapters and meta-adapters to the network, which had been trained on the DermaMNIST dataset. During meta-training, these adapters performed adaptations to either attacks or a five-class classification task, which was randomly generated from a selection of the nine-class PathMNIST set, the eight-class BloodMNIST set, or the seven-class DermaMNIST set. Subsequently, to verify the capability for rapid adaptation to a new task change, the new task was considered either as a classification task with the full set of PathMNIST classes or as a classification task with the full set of BloodMNIST classes. The resilience curve is constructed over 20 mini-batch fine-tunings, from which the resilience criterion (2) is calculated.

Taking into account the elements of randomization, it is proposed to use their average values when assessing the accuracy of the model. To this end, 100 implementations of a certain type of disturbance are generated and applied to the same model or data.

Uncertainty calibration will be performed on a dataset containing augmented test samples and out-of-distribution samples generated by Soft Brownian Offset Sampling. 300 images per class are generated for in-distribution test set to calibrate the uncertainty. The total number of out-of-distribution images is the same as the in-distribution calibration set. In this case, the Soft Brownian Offset Sampling algorithm is used with the following parameter values: minimum distance to in-distribution data is equal to 25; offset distance is equal to 20; and softness is equal to 0. Bayesian Binning into Quantiles with 10 bins was chosen as the calibration algorithm.

### 3.2 Results

Table 2 illustrates the change in accuracy with (ACC1) and without (ACC2) accounting for rejected decisions at different successive parts of the ResNet-18 model life cycle with resilience-aware add-ons under fault injections, depending on the selected MLOps configuration. Test 0, Test 1, Test 2, and Test 3 were repeated 100 times each, and Table 2 shows the average accuracy to account for the randomization effect.

**Table 2:** Accuracy of the ResNet-18-based model under the influence of fault injection during the life cycle depending on the MLOps configuration.

MLOps configuration	Test 0		Test 1		Test 2		Test 3	
	ACC1	ACC2	ACC1	ACC2	ACC1	ACC2	ACC1	ACC2
Config 0	0.751	0.781	0.652	0.679	0.659	0.681	0.623	0.634
Config 1	0.750	0.801	0.722	0.765	0.737	0.773	0.739	0.774
Config 2	0.768	0.822	0.734	0.810	0.749	0.822	0.747	0.798

Table 2 shows that after the first test with fault injection (Test1) and the last test with increasing fault injection intensity (Test3), config 1 (with resilience optimization) and config 2 (with uncertainty calibration) provide an increase in fault tolerance, which is fully consistent with the goals of resilience-aware MLOps. In addition, the dynamics of accuracy growth during adaptation (Tes 1-Test 2) is higher in the latter two configurations. Even with an increase in the fraction of damaged weight tensors, the accuracy of the system almost does not drop, unlike the configuration of conventional MLOps. Also, comparing ACC2 with ACC1, we can conclude that ACC2 is always larger than ACC1, but this difference is greater in the case of resilience optimization, and especially in the case of uncertainty calibration. Note that the averaged values of ACC1 and ACC2 for the ResNet-18-based model on Test 0-Test 3 test data with the corresponding fault injection rate without fine-tuning on 10% of human-labeled examples are 0.627 and 0.638, respectively. It proves the importance of using an active feedback loop for adaptation. For the average accuracy values in Table 2, the margin of error does not exceed 1% at a 95% confidence level.

Table 3 illustrates the change in the average accuracy with and without rejected samples, i.e., ACC1 and ACC2, at different successive parts of the lifecycle of the ResNet-18 model with resilience-aware add-ons under adversarial evasion attacks, depending on the selected MLOps configuration. Test 0, Test 1, Test 2, and Test 3 are repeated 100 times each, and Table 3 shows the average accuracy to account for the randomization effect.

**Table 3:** Accuracy values of the ResNet-18-based model under adversarial attack during the life cycle depending on the MLOps configuration.

MLOps configuration	Test 0		Test 1		Test 2		Test 3	
	ACC1	ACC2	ACC1	ACC2	ACC1	ACC2	ACC1	ACC2
Config 0	0.751	0.781	0.683	0.689	0.708	0.721	0.663	0.674
Config 1	0.750	0.801	0.735	0.785	0.749	0.797	0.742	0.753
Config 2	0.768	0.822	0.753	0.810	0.770	0.847	0.768	0.802

The results of Test 1 and Test 3 in Table 3 show that config 1 (with resilience optimization) and config 2 (with uncertainty calibration) provide an increase in robustness. In addition, the dynamics of accuracy growth during adaptation (Tes 1, Test 2) is higher in the latter two configurations. However, the traditional MLOps (config 0) also showed the ability to adapt quickly during fine-tuning (comparing the results of Test 1 and Test 2), although it was not successful in restoring performance. Config 1 and Config 2 show a noticeable recovery in accuracy, and config 2 on Test 2 even showed an improvement in accuracy compared to its pre-disturbance value. Increasing the magnitude of the perturbation (Test 3) leads to a decrease in accuracy in all cases, but config 1 and config 2 show greater resilience compared to config 0. Also, across all experiments, ACC2 is larger than ACC1, which indicates the ability to recognize disturbances that cannot be absorbed. Note that the averaged values of ACC1 and ACC2 for the ResNet-18-based model on perturbed test data from Test 0-Test 3 stages without fine-tuning on 10% of human-labeled examples are 0.671 and 0.682, respectively. It also proves the importance of using an active feedback loop for adaptation. For the average accuracy values in Table 3, the margin of error does not exceed 1.1% at a 95% confidence level.

Tables 4, 5 illustrate the changes in the accuracy values of the MedViT-T-based model under the influence of fault-injection and adversarial attack during the life cycle, depending on the MLOps configuration. In the MedViT-T experiments, Test 0, Test 1, Test 2, and Test 3 are repeated 100 times each, and the average accuracy is reported in Tables 4, 5.

**Table 4:** Accuracy values of the MedViT-T-based model under the influence of fault injection during the life cycle depending on the MLOps configuration.

MLOps configuration	Test 0		Test 1		Test 2		Test 3	
	ACC1	ACC2	ACC1	ACC2	ACC1	ACC2	ACC1	ACC2
Config 0	0.769	0.791	0.672	0.698	0.671	0.751	0.633	0.694
Config 1	0.772	0.835	0.742	0.775	0.750	0.781	0.731	0.784
Config 2	0.777	0.902	0.752	0.820	0.759	0.842	0.750	0.808

**Table 5:** Accuracy values of the MedViT-T-based model under the influence of adversarial attack during the life cycle depending on the MLOps configuration.

MLOps configuration	Test 0		Test 1		Test 2		Test 3	
	ACC1	ACC2	ACC1	ACC2	ACC1	ACC2	ACC1	ACC2
Config 0	0.769	0.791	0.705	0.748	0.710	0.751	0.697	0.750
Config 1	0.772	0.835	0.742	0.788	0.750	0.781	0.748	0.780
Config 2	0.777	0.902	0.760	0.833	0.767	0.842	0.759	0.800

An analysis of Tables 4, 5 shows that MedViT-T exhibits similar behavior to ResNet-18 on the same tests and MLOps configurations. However, MedViT-T is characterized by a lower adaptation rate (comparison of Test1, Test2 results) compared to ResNet-18. Note that the averaged values of ACC1 and ACC2 for the MedViT-T-based model on Test 0-Test 3 test data with the corresponding fault injection rate without fine-tuning on 10% of human-labeled examples are 0.653 and 0.694, respectively. It proves the importance of using an active feedback loop for adaptation. Averaged values of ACC1 and ACC2 for the MedViT-T-based model on perturbed test data from Test 0 to Test 3 stages without fine-tuning on 10% of human-labeled examples are 0.687 and 0.695, respectively. It also proves the importance of using an active feedback loop for adaptation. For the average accuracy values presented in Table 4, the margin of error does not exceed 0.9% at a 95% confidence level. Similarly, for the average accuracy values in Table 5, the margin of error does not exceed 1% at the same confidence level.

To evaluate the robustness and speed of adaptation of a pre-configured medical AI system to concept drift, it is proposed to calculate the integral resilience criterion (2) in fine-tuning mode (few-shot learning) on BloodMNIST dataset or PathMNIST dataset (Table 6). In this case, the AI system was pre-trained and optimized on the DermaMNIST set. It is proposed to use 16 images per class (k_shot = 16), which are provided in mini-batches of four images (mini_batch_size = 4) during adaptation.

**Table 6:** The value of the integral resilience criterion (2) to the change of the medical image analysis task depending on the MLOps configuration.

MLOps configuration	Fine-tuning of ResNet-18-based AI model		Fine-tuning of MedViT-T-based AI model	
	On 20 mini-batches of BloodMNIST (complete set of classes)	On 20 mini-batches of PathMNIST (complete set of classes)	On 20 mini-batches of BloodMNIST (complete set of classes)	On 20 mini-batches of PathMNIST (complete set of classes)
Config 0	0.68	0.74	0.66	0.71
Config 1	0.80	0.82	0.71	0.74

Analysis of Table 6 shows that adding a resilience optimization stage to MLOps increases resilience to concept drift, i.e., robustness and speed of adaptation. However, the architecture of visual transformers in our experiments shows itself to be less resilient. For the average accuracy values in Table 6, the margin of error does not exceed 1% at a 95% confidence level.

## 4 Discussion

The proposed structure of resilience-aware MLOps makes it possible to implement various specific solutions for the implementation of its separate stages and mechanisms. The main idea is to divide the labor of developers of the basic AI model that functions efficiently under normal conditions and specialists in ensuring the resilience of the intelligent system to disturbances and changes. The healthcare industry is extremely complex and requires deep knowledge in various fields. Developers of the basic AI model are usually overloaded with taking into account the specifics of data, methods of data collection and the application itself to solve the applied data analysis task. Solving problems related to ensuring AI resilience, i.e., security, trustworthiness, robustness and rapid adaptation to changes, relies on specific expertise not related to a particular application area (13). The main difficulty in separating the tasks is the lack of universality of methods for ensuring resilience and the lack of a complete understanding of the compatibility of methods that provide different aspects of resilience and protection against different types of disturbances (16). Determining the compatibility of methods and combining them can increase flexibility and resilience depending on requirements and constraints.

The proposed implementation of *Post-hoc* Resilience Optimization is just one of the possible solutions that shows the fundamental possibility of separating the stage of developing a basic model for normal conditions and add-ons to ensure resilience to disturbances and changes. Moreover, the importance of using the proposed *Post-hoc* Uncertainty Calibration stage was experimentally confirmed. This stage allows, firstly, to detect disturbances and, secondly, to correctly assess uncertainty and tolerate it, i.e., to ensure trustworthiness.

Unexpectedly, the worse resilience indicators for the visual transformer compared to the convolutional network were found. This indicates that there is a need to improve architectures and connection methods for adapters and meta-adapters. Similarly, uncertainty calibration methods did not ensure 100% accuracy of the models. This is partly due to the fact that there are two types of uncertainty—aleatory and epistemic uncertainty. In this case, the aleatory uncertainty cannot be eliminated, because it is an inherent part of the observed process or object (46). On the other hand, there are many calibration algorithms, each of which depends on a large number of hyperparameters. Understanding their impact on epistemic uncertainty in the context of Resilient-aware MLOps is an important research area. Progress in AI architectures and their hybridization also requires the development of a methodology to increase the flexibility of Resilience-aware MLOps tools.

Besides, it is needed to underline the following. The resilience of AI systems should, on the one hand, take into account traditional models and principles (47, 48) of its provision, which are based on evolutionary mechanisms for taking into account and adapting to changes in requirements, parameters of the information and physical environments, tolerance to uncertain failures, taking into account cyberattacks (49, 50). On the other hand, as it was mentioned in (16) the actual models and means of artificial intelligence have the potential of natural resilience, which should be used and which is used. This study is the next step in improving both of these approaches. From the point of view of the general principles of resilience, the idea of a certain resilient wrapper is proposed, which can be adapted for different applications. With regard to the developing the methodology of naturally resilient AI, the proposed solutions can be further improved through a more flexible setting, taking into account the features of the functional part of AI.

## 5 Conclusion

### 5.1 Summary

The structure of resilience-aware MLOps for medical diagnostic systems has been proposed. The main novelty lies in the separate work on creating a basic model for normal operating conditions and work on ensuring its resilience and trustworthiness. This is significant for the medical industry, as the developer of the basic model should devote more time to comprehending medical field and the diagnostic task at hand, rather than specializing in a specific area of resilient systems. Therefore, *Post-hoc* Resilience Optimization, *Post-hoc* Predictive Uncertainty Calibration, Uncertainty Monitoring and Graceful Degradation are used as additional stages of MLOps.

Resilience optimization increases robustness to disturbances and speed of adaptation. Fault injection attack, adversarial evasion attack, and concept drift are considered as disturbances. In order to optimize the resilience of the AI-based disease recognition system, additional add-ons are used in the form of adapters and meta-adapters. Meta-adapters are fine-tuned during meta-training based on the results of adaptation to synthetic disturbances. An additional model is added for *Post-hoc* Calibration of Predictive Uncertainty, which is tuned on in-distribution data and out-of-distribution data, to correct predictive confidence in inference mode. Calibrated confidences ensures recognition of a part of unabsorbed disturbances to mitigate their influence, and it improves the trustworthiness.

Experimentally confirmed increase of robustness and speed of adaptation for medical image recognition system during several intervals of the system’s life cycle due to the use of resilience optimization and uncertainty calibration stages. The experiments were performed on the DermaMNIST dataset, BloodMNIST and PathMNIST. ResNet-18 as a representative of convolutional networks and MedViT-T as a representative of visual transformers are considered (51). It is shown that transformers are less resilient than a convolutional network, but this may be due to the imperfect architecture of adapters and meta-adapters.

### 5.2 Limitation

This study is demonstrated on the example of a medical image classification system and does not describe the specifics of using resilience-aware MLOps for self-supervised or reinforcement learning systems. Nevertheless, the general framework of resilience-aware MLOps can be applied to every type of intelligent system. Another limitation may be related to attempts to generalize the information found, which may affect the completeness of the literature review.

Moreover, well-known approaches to Explainable artificial intelligence, as well as Graceful Degradation, are excluded from detailed analysis of their impact on resilience. The paper focuses on the analysis of the general structure of resilience-aware MLOps and the stages of resilience optimization and predictive uncertainty calibration.

### 5.3 Future research direction

Future research should focus on the development new flexible adapter and meta-adapter architectures as addons for AI system resilience. Special attention should also be paid to the question of providing resilience for self-supervised and reinforcement learning systems. Another important area of research should be the investigation of methods to ensure resilience to new types of attacks on AI systems in the healthcare industry.

## Data availability statement

Publicly available datasets were analyzed in this study. This data can be found at: https://zenodo.org/record/6496656#.ZGMJ--xByys.

## Author contributions

VM: Writing – original draft. VK: Supervision, Writing – review & editing.
